# Supplementary material for: Causal interplay between lactose intolerance and gut microbiota: a combined bidirectional Mendelian randomization and in vivo validation study
Source: Front Nutr. 2026 Jun 1;13:1803337. doi: 10.3389/fnut.2026.1803337 (PMC13265576; doi:10.3389/fnut.2026.1803337)
Supplement: Supplementary file 1 [file Data_Sheet_1.zip › supplementary materials/Forward/leave one out cross validation/ebi-a-GCST90027488.finngen_R12_E4_LACTONAS.pdf]

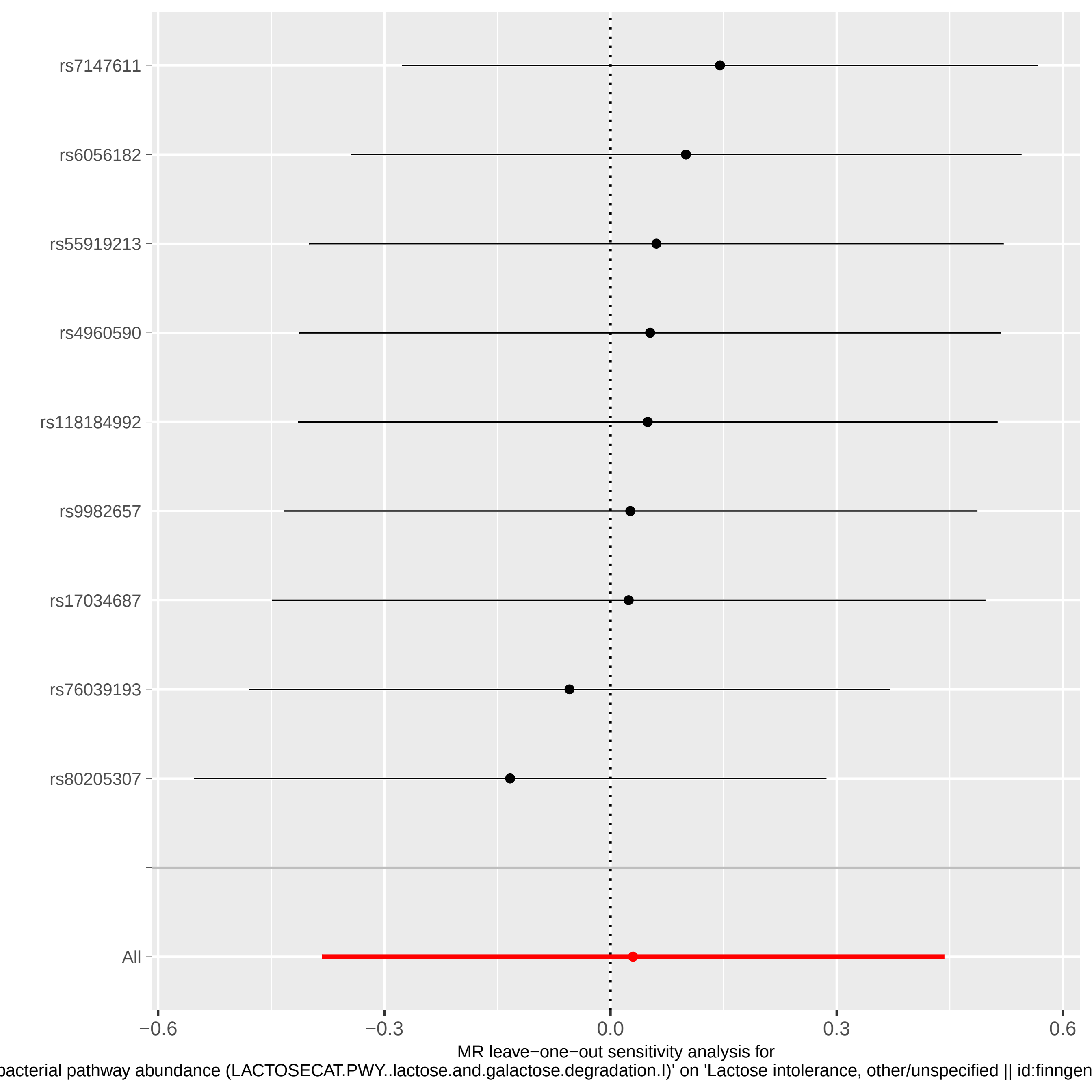
rs7147611
rs6056182
rs55919213
rs4960590
rs118184992
rs9982657
rs17034687
rs76039193
rs80205307
All
−0.6
−0.3
0.0
0.3
0.6
MR leave−one−out sensitivity analysis for
bacterial pathway abundance (LACTOSECAT.PWY..lactose.and.galactose.degradation.I)' on 'Lactose intolerance, other/unspecified || id:finngen
